# Supplementary material for: Polyurea–Graphene Nanocomposites—The Influence of Hard-Segment Content and Nanoparticle Loading on Mechanical Properties
Source: Polymers (Basel). 2023 Nov 16;15(22):4434. doi: 10.3390/polym15224434 (PMC10675114; doi:10.3390/polym15224434)
Supplement: Supplementary file 1 [file polymers-15-04434-s001.zip › polymers-2675784-supplementary.pdf]

Supplementary Materials for:

Polyurea-Graphene Nanocomposites – the Influence of Hard-Segment Content and Nanoparticle Loading on Mechanical Properties

Demetrios A. Tzelepis [1], Arman Khoshnevis [2], Mohsen Zayernouri [3] and Valeriy V. Ginzburg [4*]

[1] Department of Chemical Engineering and Materials Science, Michigan State University, East Lansing, Michigan, USA; email tzelepi1@msu.edu
[2] Department of Mechanical Engineering, Michigan State University, East Lansing, Michigan, USA; email khoshne1@msu.edu
[3] Department of Mechanical Engineering, Michigan State University, East Lansing, Michigan, USA; email zayern@msu.edu
[4] Department of Chemical Engineering and Materials Science, Michigan State University, East Lansing, Michigan, USA; email ginzbur7@msu.edu
* Correspondence: ginzbur7@msu.edu

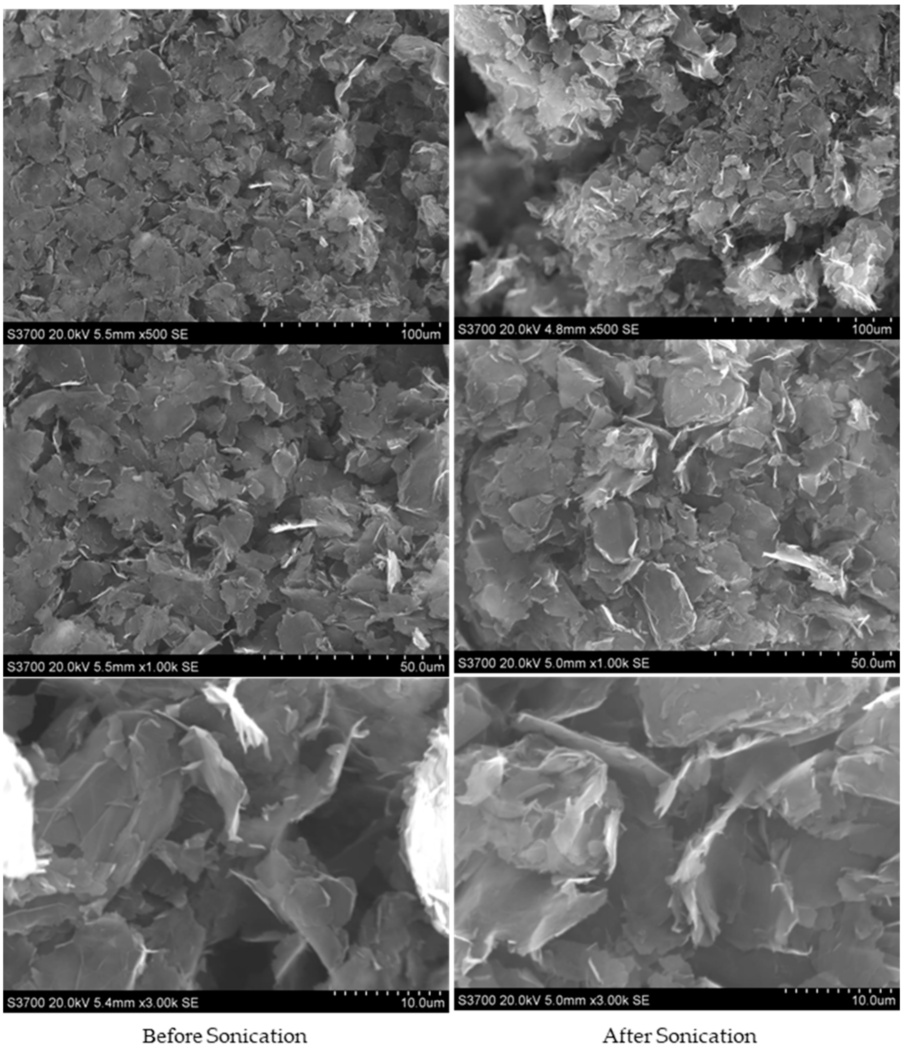

Figure S1 - SEM images at various magnifications of the xGnP before (left column) and after sonication (right column). The images indicate that there is no change in the morphology of the xGnP with the sonication parameters used.

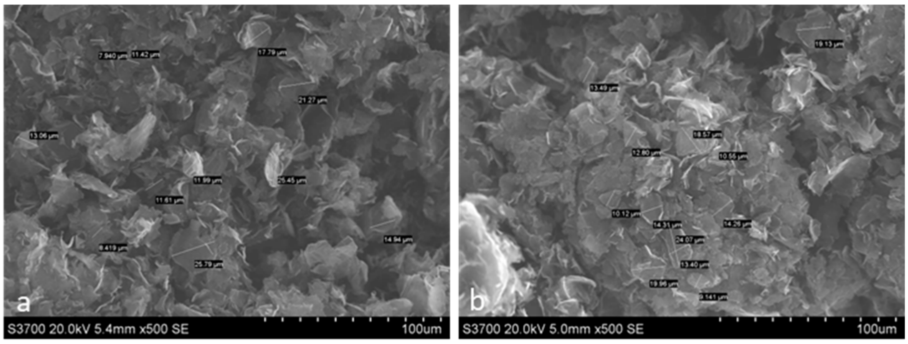

Figure S2 - SEM micrographs showing the measurement of the estimated diameter of the xGnP before sonication (a) and after sonication (b).

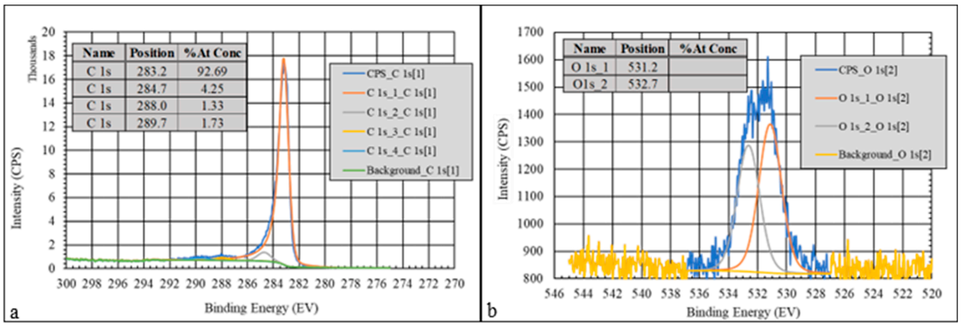

Figure S3 – Deconvolution of the XPS spectrum, for the heat-treated xGnP, in the binding energy region for C (a), and O (b).

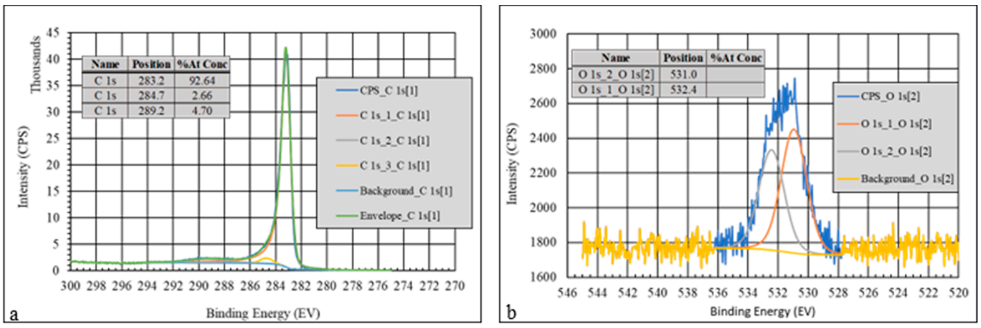

Figure S4 - Deconvolution of the XPS spectrum, for the heat-treated and sonicated xGnP and sonicated, in the binding energy region for C (a), and O (b).

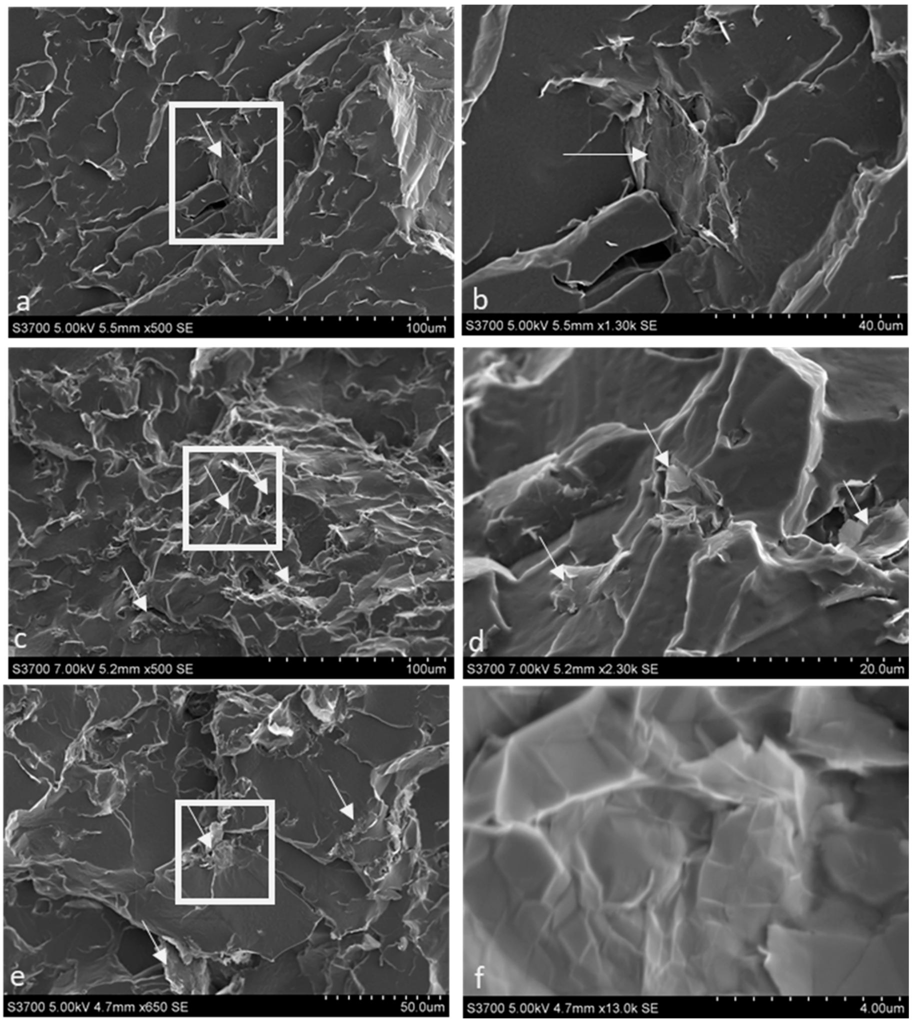

Figure S5 - SEM photomicrographs of the fracture surface for all IPDI-2k-20HS xGnP loadings: a) 0.5 wt% xGnP loading at 500x, b) Photomicrograph of the white box in a. c) 1.0 wt% xGnP loading at 500x, d) Photomicrograph of the white box in c. e) 1.5 wt% xGnP loading; f) Photomicrograph of the white box in e.

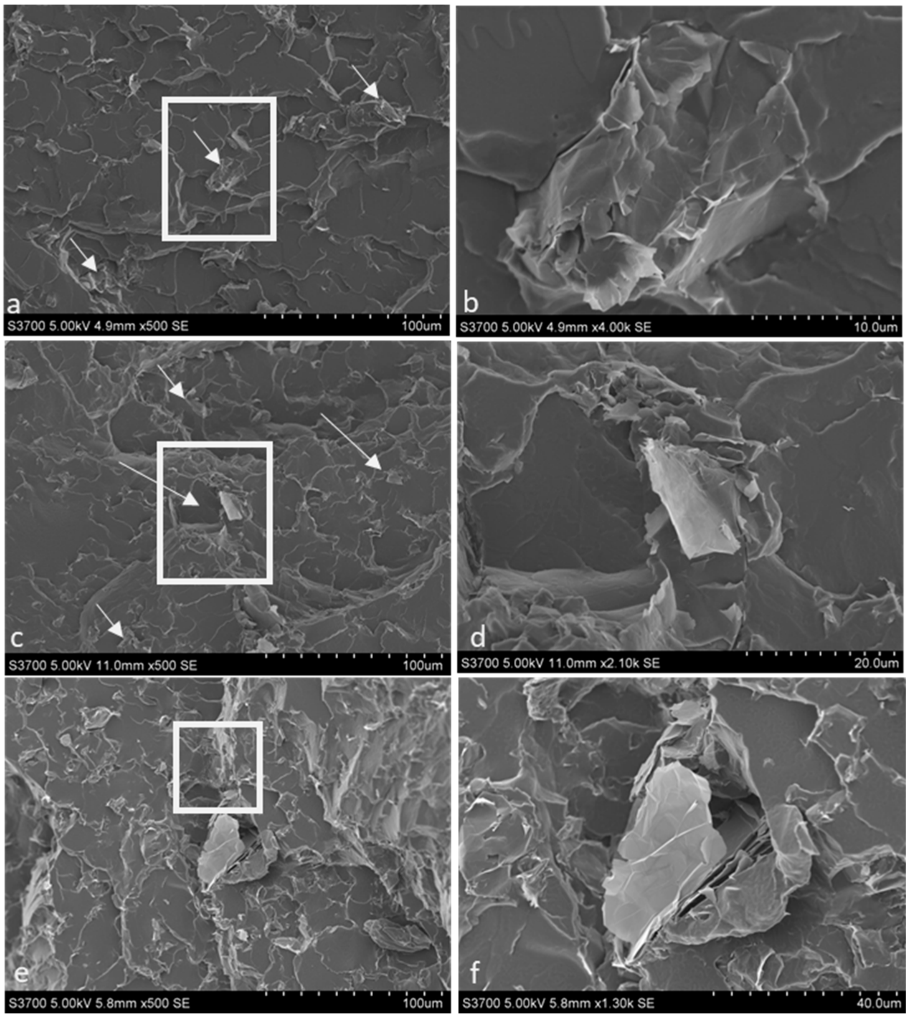

Figure S6 - SEM photomicrographs of the fracture surface for all IPDI-2k-30HS xGnP loadings: a) 0.5 wt% xGnP loading at 500x, b) Photomicrograph of the white box in a. c) 1.0 wt% xGnP loading at 500x, d) Photomicrograph of the white box in c. e) 1.5 wt% xGnP loading at 650x. f) Photomicrograph of the white box in e. In all photomicrographs the arrows point to the xGnP.

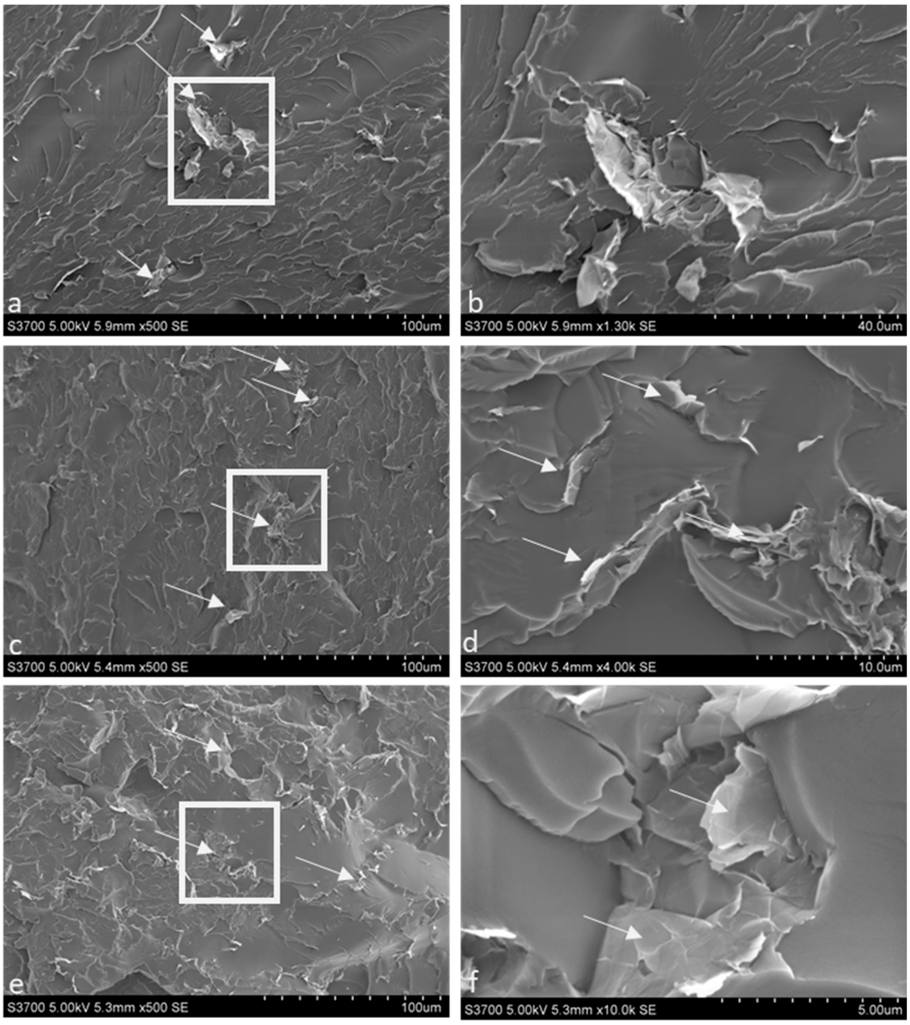

Figure S7 - SEM photomicrographs of the fracture surface for all IPDI-2k-40HS xGnP loadings: a) 0.5 wt% xGnP loading at 500x, b) Photomicrograph of the white box in a. c) 1.0 wt% xGnP loading at 500x, d) Photomicrograph of the white box in c. e) 1.5 wt% xGnP loading at 650x. f) Photomicrograph of the white box in e. In all photomicrographs the arrows point to the xGnP.

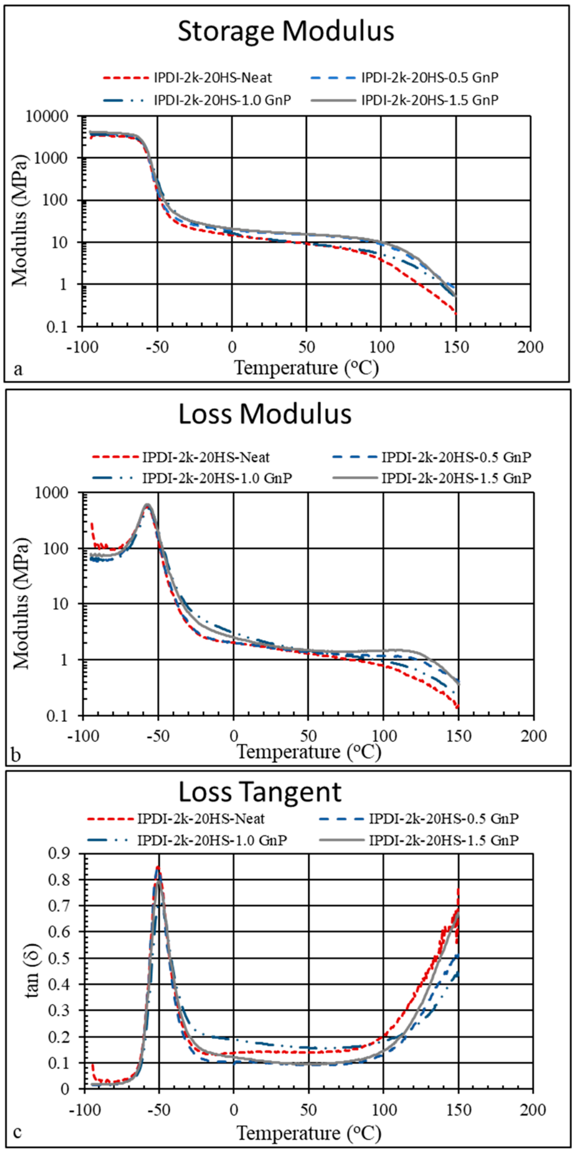

Figure S8 – DMA temperature sweep showing the storage, loss modulus and the tan (δ) for the IPDI-2k-20HS.

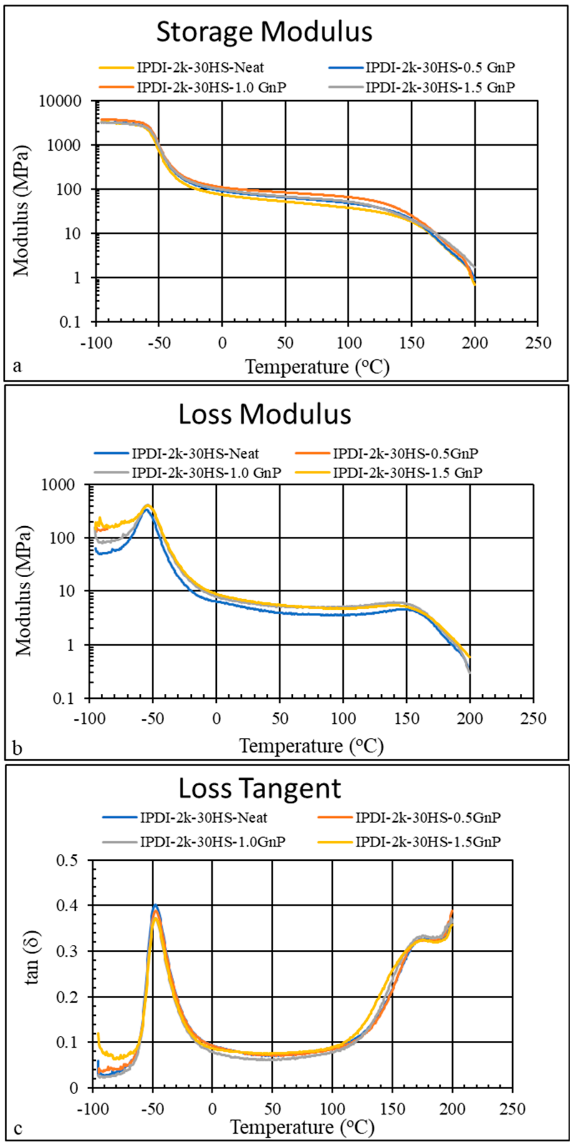

Figure S9 – DMA temperature sweep showing the storage, loss modulus and the tan (δ) for the IPDI-2k-30HS.

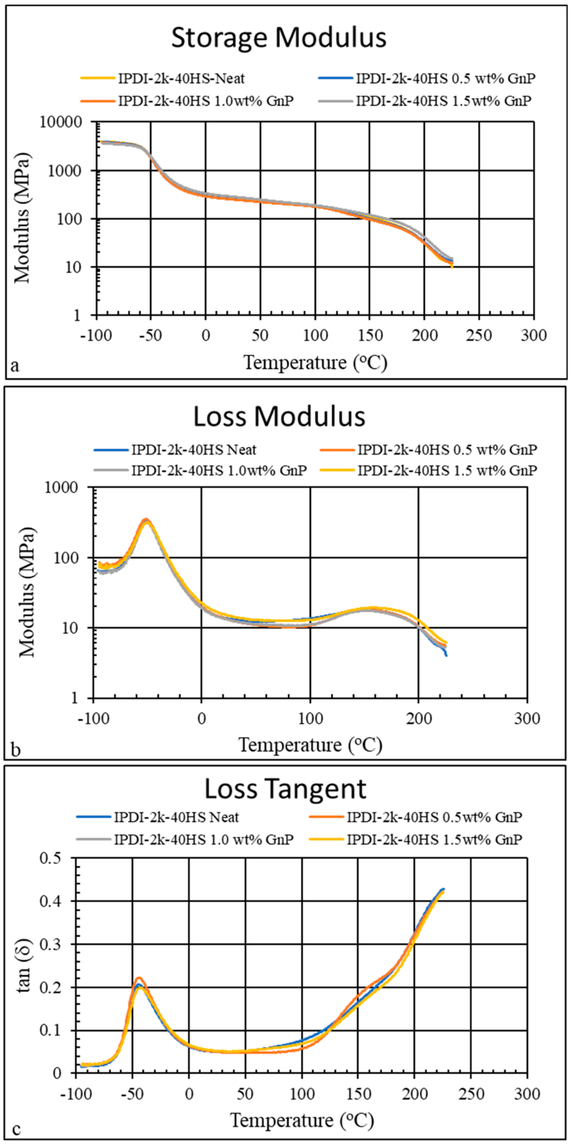

Figure S10 – DMA temperature sweep showing the storage, loss modulus and the tan (δ) for the IPDI-2k-40HS.

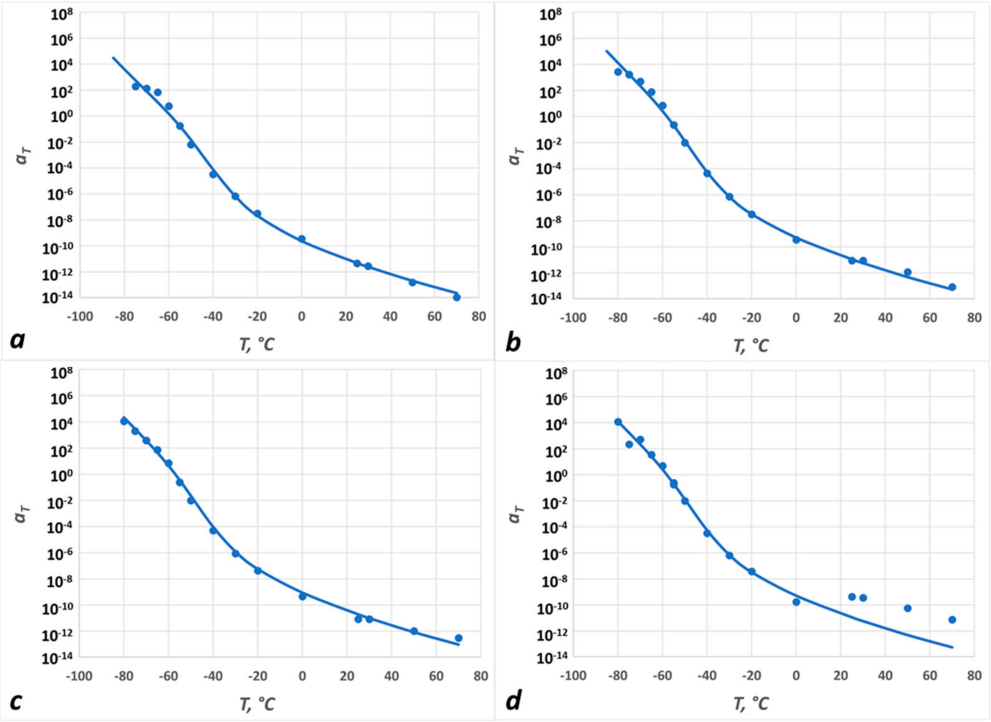

Figure S11: Experimental (symbols) and TS2 fit (lines) shift factors for IPDI-2k-30HS polyureas with: (a) No added nanofillers; (b) 0.5 wt % xGnP; (c) 1.0 wt % xGnP; (d) 1.5 wt % xGnP.

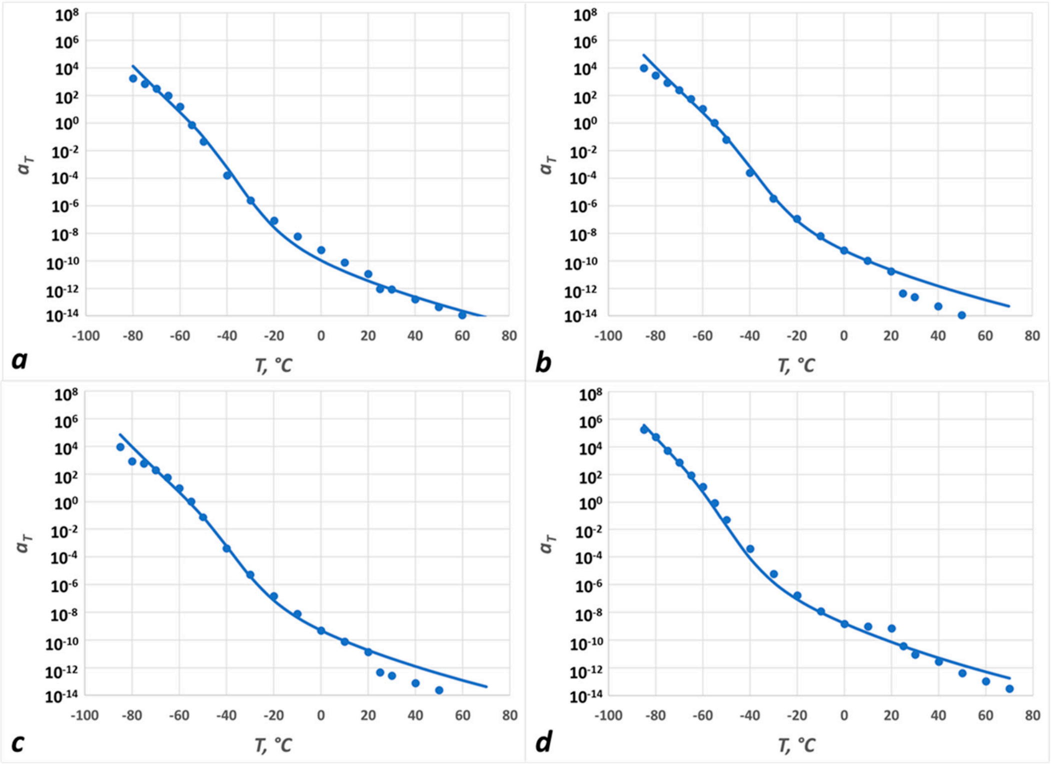

Figure S12: Experimental (symbols) and TS2 fit (lines) shift factors for 40HS polyureas with: (a) No added nanofillers; (b) 0.5 wt % xGnP; (c) 1.0 wt % xGnP; (d) 1.5 wt % xGnP

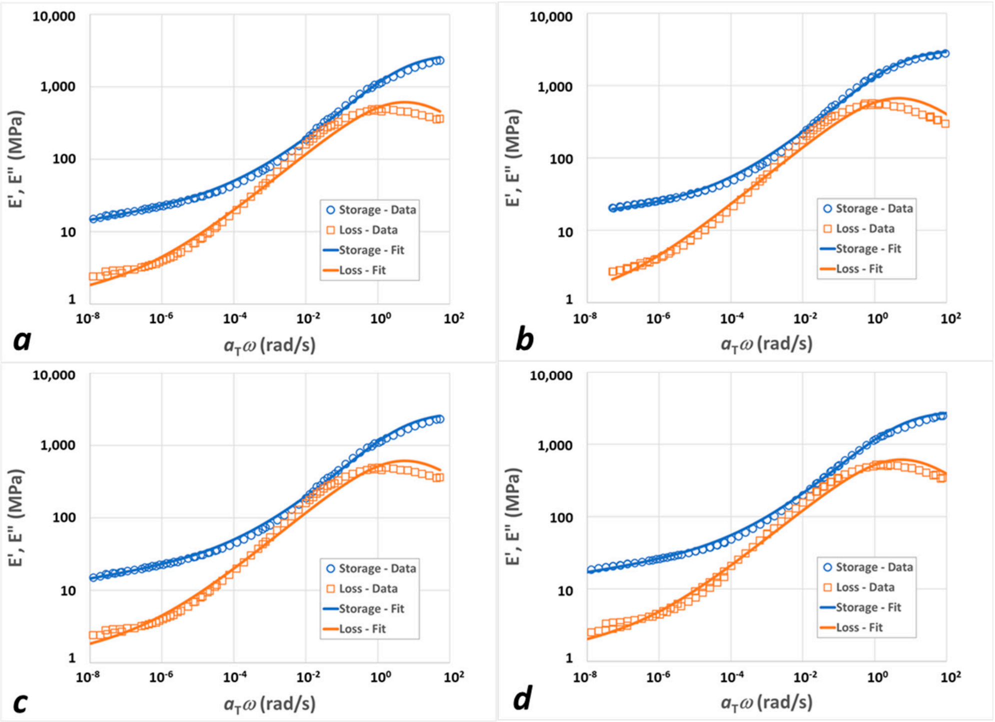

Figure S13: Experimental (symbols) and FMG-FMG fit (lines) master curves for IPDI-2k-20HS polyureas with: (a) No added nanofillers; (b) 0.5 wt % xGnP; (c) 1.0 wt % xGnP; (d) 1.5 wt % xGnP. Blue open circles represent storage modulus data, blue lines are the storage modulus model fits; orange open squares correspond to the loss modulus data, and orange lines are the loss modulus model fits

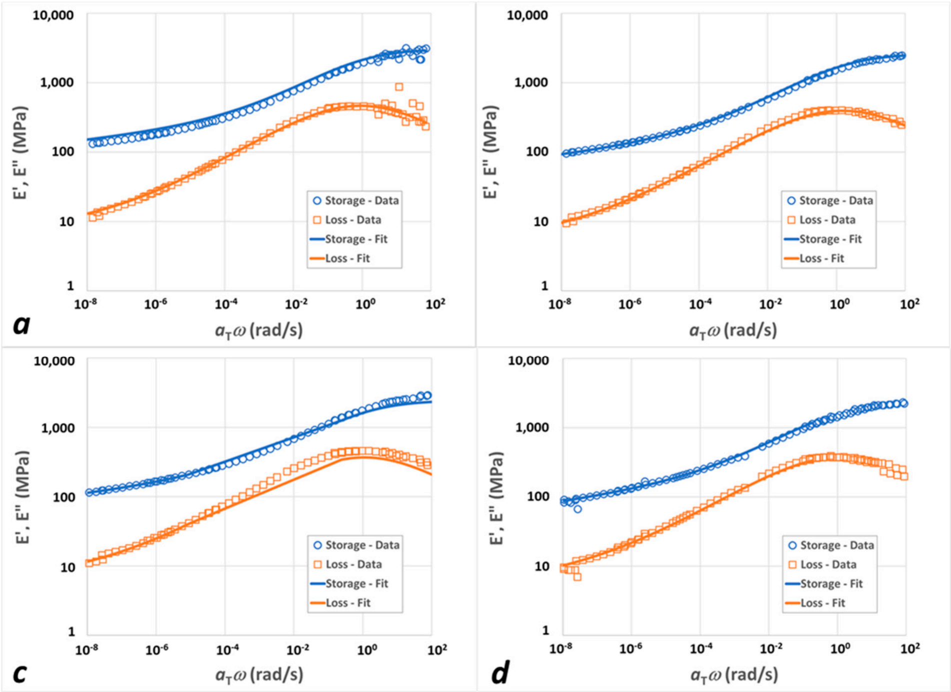

Figure S14: Experimental (symbols) and FMG-FMG fit (lines) master curves for 30HS polyureas with: (a) No added nanofillers; (b) 0.5 wt % xGnP; (c) 1.0 wt % xGnP; (d) 1.5 wt % xGnP. Blue open circles represent storage modulus data, blue lines are the storage modulus model fits; orange open squares correspond to the loss modulus data, and orange lines are the loss modulus model fits

Table S1: TTS reference temperatures and TS2 fit parameters for all systems

Polymer	%xGnP	T_0, K	E_1, kJ/mol	E_2, kJ/mol	$\Delta S/R$	T*, K
IPDI-2k-20HS	0.00%	213	94.5	120.0	25.0	217.1
	0.50%	214	94.5	121.1	25.0	218.5
	1.00%	217	94.5	118.8	25.0	222.5
	1.50%	215	94.5	116.5	25.0	222.8
IPDI-2k-30HS	0.00%	214	99.9	120.0	25.0	233.8
	0.50%	215	99.9	120.6	25.0	228.9
	1.00%	216	99.9	120.6	25.0	228.9
	1.50%	215	99.9	120.6	25.0	228.9
IPDI-2k-40HS	0.00%	217	100.0	123.6	25.0	240.5
	0.50%	218	100.0	120.4	25.0	238.5
	1.00%	217	100.0	120.4	25.0	238.5
	1.50%	216	100.0	120.8	25.0	225.6
